# A fast and easy-to-perform noninvasive Muller’s muscle sublimation technique using plasma technology for treatment of mild-to-moderate ptosis: a case report

**DOI:** 10.1186/s13256-025-05330-y

**Published:** 2025-06-17

**Authors:** Nasrin Raffati

**Affiliations:** Negah Aref Ophthalmic Research Center, Negah Eye Hospital, Tehran, Iran

**Keywords:** Ptosis, Muller’s muscle, Noninvasive, Plasma

## Abstract

**Background:**

Currently, most surgeons favor Muller’s muscle conjunctival resection as a treatment for mild-to-moderate ptosis, as this method is associated with fewer post-surgical complications.

**Case presentation:**

This case series of six Iranian women aged between 15 and 65 (mean 43) years with mild-to-moderate ptosis treated at Negah Eye Hospital used a plasma scalpel (PLEXR™) for conjunctivo-Muller sublimation. A 1–4 area management strategy was applied, and margin reflex distance 1 measurements showed improvement from 1.58 mm pre-treatment to 3.66 mm after 6 months.

**Conclusion:**

Seemingly, applying plasma technology to treat mild-to-moderate ptosis via conjunctivo-Muller muscle sublimation might be a promising alternative to more invasive surgical methods.

## Introduction

Ptosis, also called blepharoptosis, is a common ophthalmic condition characterized by an abnormally low-positioned upper eyelid [1]. Ptosis presents multifaceted challenges to patients, ranging from compromised aesthetics to functional impairments affecting vision and overall quality of life [2].

Most ptosis treatment methods involve either resecting and advancing or plicating the levator muscle through an anterior approach or the Muller’s muscle through a posterior approach. Traditionally, the more common surgical method has been external levator advancement [3, 4]. Unfortunately, this method is associated with many complications such as poor contour and lid peaking; dry eye syndrome; corneal abrasion; undercorrection or overcorrection causing eyelid asymmetry and cosmetic challenges; lagophthalmos; and scleral show [5, 6]. Currently, most surgeons favor Muller’s muscle conjunctival resection as a treatment for mild-to-moderate ptosis, as this method is associated with fewer post-surgical complications [7, 8].

In recent years, plasma-assisted treatment has emerged as a promising alternative to surgical intervention in various ocular disorders such as conjunctival nevus [9], pinguecula [10], punctal occlusion [11], conjunctival cyst, [12], and blepharochalasis [13–17]. This method utilizes the power of low-temperature plasma to achieve precise tissue sublimation and shrinkage without the need for incisions or sutures [18]. The noninvasive nature of this method is particularly appealing for patients seeking minimally disruptive solutions [15].

This case series aims to evaluate using plasma-assisted modality applied directly to conjunctivo-Muller’s muscle as a novel technique for minimal ptosis correction using plasma technology. The study’s goal is to evaluate this new approach’s effectiveness and potential limitations, which promises to be a quicker, simpler, and less invasive treatment option for a small group of patients. To the best of our knowledge, the present report is the first report on using plasma technology applied directly to conjunctivo-Muller’s muscle to treat ptosis.

## Case presentation

This case series included six Iranian women aged between 15 and 65 (mean 43) years with ptosis who came to Negah Eye Hospital, Tehran, Iran, from May 2022 to September 2023. The criteria for including patients in the study were as follows: they had to be older than 15 years; have no history of previous intraorbital and strabismus operations or ptosis surgery using the posterior approach; and have a normal conjunctiva. They also needed to be willing to complete a 6-month follow-up. In two cases, patients tested positive for phenylephrine. Prior to treatment, patients’ demographic profiles, ptosis etiologies, levator muscle function, and presence of preoperative lagophthalmos were recorded.

Follow-ups were carried out on day 1, day 5 (for extracting the bandage contact lens), 1 month, and 6 months post-surgery. The postoperative margin reflex distance 1 (MRD1) values were recorded at 6-month follow-ups.

The MRD1 values were obtained by directing light from a 1 m distance in front of the patient’s corneas. A photograph was captured at this moment, and the measurement in millimeters from the upper lid margin to the corneal light reflex in the image was designated as the MRD1. The patients were seated, and their eyes were in the primary gaze. The patients were classified into three groups according to their preoperative MRD1: mild ptosis (MRD1 1–2 mm), moderate ptosis (MRD1 2–3 mm), and severe ptosis (MRD1 ≥ 3 mm). Furthermore, patients were asked before and after surgery about changes in dry eye symptoms, including eye strain and tears.

This study complies with the ethical standards of the relevant national and institutional committees on human experimentation and with the Helsinki Declaration of 1975, as revised in 2013. In addition, the authors assert that all procedures contributing to this study adhere to the ethical standards of the relevant national and institutional guides on the care and use of laboratory animals.

### Surgical technique

Patients were first photographed after entering the operation room for a preoperation photo to compare with postoperative results. In the following step, patients underwent either general anesthesia or a combination of intravenous sedation and local lidocaine 2% injection. Then, the area of plasma application was calculated using the 1–4 area management approach (4 mm conjunctiva sublimation for each 1 mm of desired ptosis correction) and subsequently, the plasma sublimation on the eyelid conjunctiva and Muller muscles of the eye with ptosis using the plasma scalpel (PLEXR™; GMV Srl., Rome, Italy) with redhead configuration (V peak-to-peak, 700 V; power, 2 W; frequency, 75 kHz). The eyelid was everted using a Desmarres retractor and after marking of conjunctiva right above superior border of tarsal plate, surgery was initiated.

**Fig. 1:**
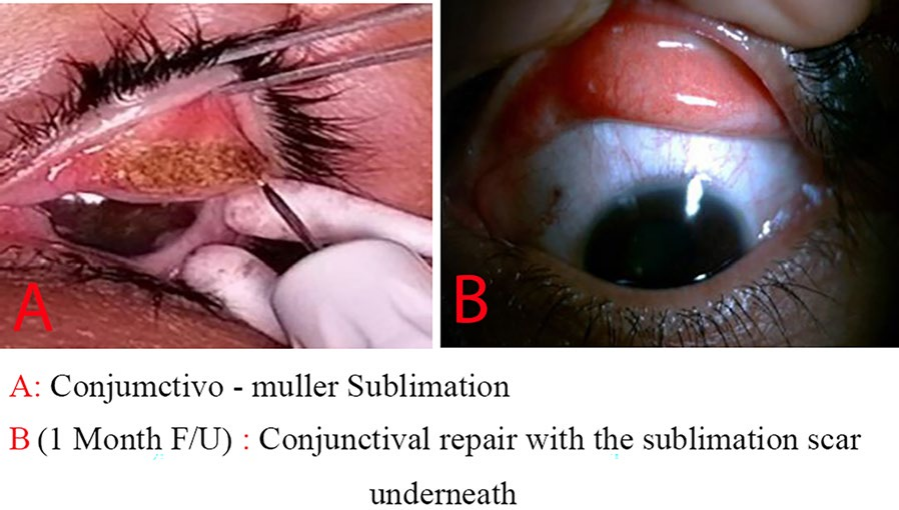
**A**, **B** Applying the sublimation directly to the conjunctivo-Muller muscle to shorten its length

In the case of intravenous sedation, local anesthesia was administered with an injection of 2 mL lidocaine 2% subconjunctively. The conjunctiva almost always remains dry, and the device’s head is used at a short distance from its surface to generate plasma. The surgeon applies a continuous spot until the sublimation of Muller’s muscle is completed (Fig. 1), with each eyelid treatment taking less than 5 minutes. Following the procedure, a bandage contact lens is applied.

This study briefly describes cases and treatment results after plasma-assisted Muller’s muscle sublimation. These results are summarized in Table 1.

**Table 1:** Summary of cases entering the study and treatment results

Patient	Eye	Age (years)	The reason for treatment	MRD1 before (mm)	LMF before (mm)	MRD1 after (mm)	LMF after (mm)
Case 1	Left	34	Ptosis in the left eye following lung surgery	1	14	3	14
Case 2	Left	15	Congenital ptosis	0	6	3	6
Case 3	Right	65	Levator muscle disinsertion from previous surgical ocular treatment	2	16	4	16
Case 4	Left	41	Congenital ptosis	2	16	4	16
Case 5	Left	54	Congenital ptosis	1	15	3	15
Case 6	Left	49	Congenital ptosis	3.5	14	5	14

*MRD1* margin reflex distance 1, *LMF* levator muscle function

### Case 1

The first patient was a 34-year-old Iranian woman with a history of ptosis in the left eye following lung surgery. She had not undergone surgical treatment for her ptosis for 2 years. MRD1 before treatment was 1 mm, and levator muscle function was 14 mm. The MRD1 improved to 3 mm, and levator muscle function remained at 14 mm. The treatment for this case was performed in two separate sections.

### Case 2

The second patient was a 15-year-old Iranian girl with a history of two levator resection surgeries in her left eye due to congenital ptosis. MRD1 before treatment was 0 mm, and levator muscle function was 6 mm. After treatment, the MRD1 improved to 3 mm, and the levator muscle function remained at 6 mm. The treatment for this case was performed in two separate sections.

### Case 3

**Fig. 2:**
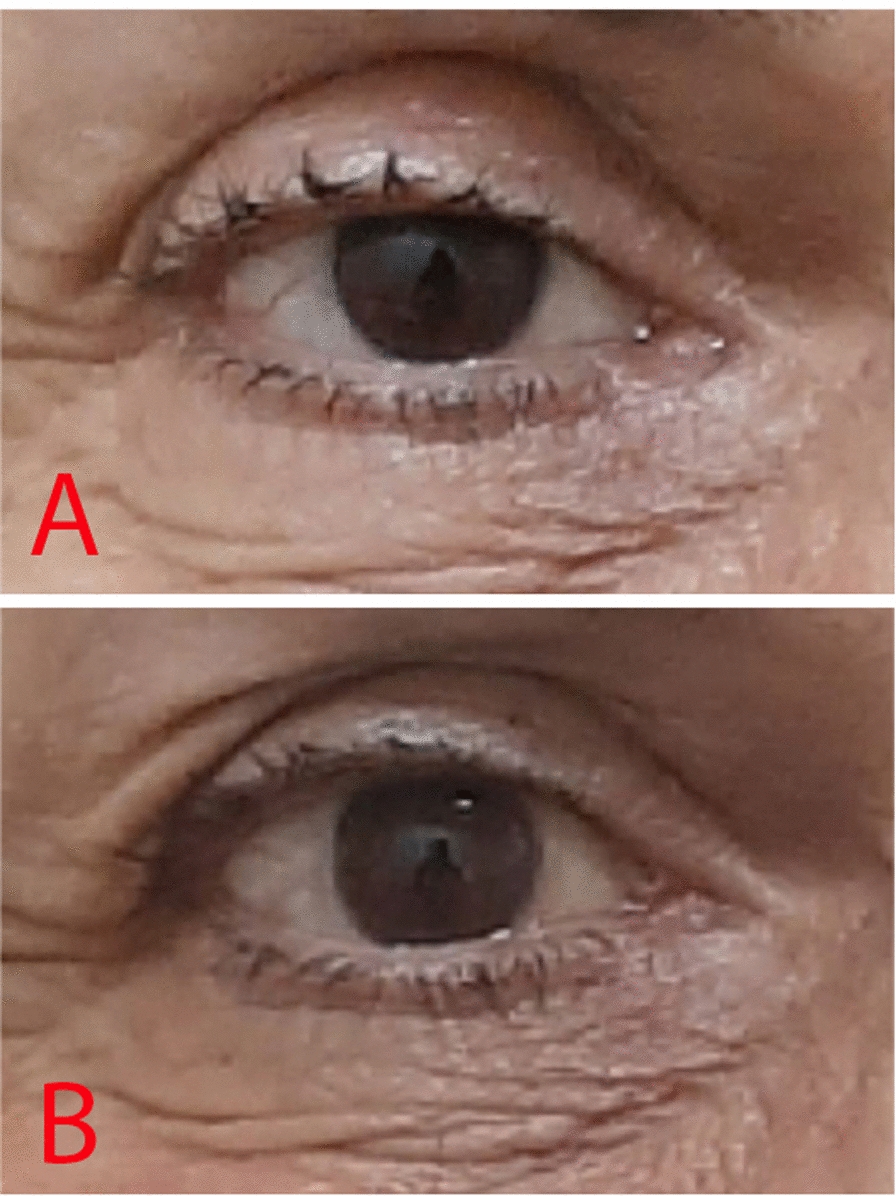
**A** The patient before treatment. Note the ptosis in the right eye. **B** Improved ptosis in the right eye after treatment

The third patient was a 65-year-old Iranian woman with a history of levator muscle disinsertion after cataract surgery in her right eye. MRD1 before treatment was 2 mm, and levator muscle function was 16 mm. After treatment, the MRD1 improved to 4 mm, and the levator muscle function remained at 16 mm (Fig. 2). Furthermore, she had a past medical history of thyroid eye diseases and lid retractors.

### Case 4

The fourth patient was a 41-year-old Iranian woman with a history of mild congenital ptosis in her left eye. Before treatment, MRD1 was 2 mm, and levator muscle function was 16 mm. After treatment, MRD1 improved to 4 mm, and levator muscle function remained at 16 mm.

### Case 5

**Fig. 3:**
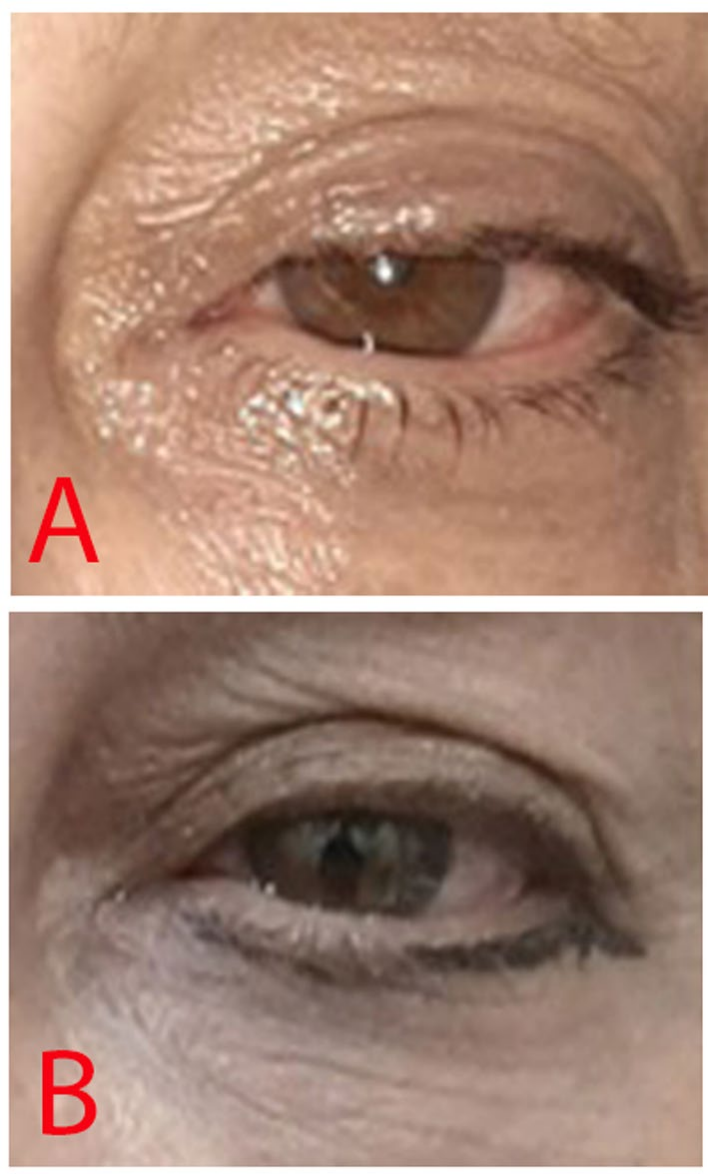
**A** The patient before treatment. Note the ptosis in the left eye. **B** Improved ptosis in the left eye after treatment

The fifth patient was a 54-year-old Iranian woman with a history of congenital ptosis in her left eye. Before treatment, MRD1 was 1 mm, and levator muscle function was 15 mm. After treatment, MRD1 improved to 3 mm, and levator muscle function remained at 15 mm (Fig. 3).

### Case 6

The sixth patient was a 49-year-old Iranian woman with a history of congenital ptosis in her left eye. Before treatment, MRD1 was 3.5 mm, and levator muscle function was 14 mm. After treatment, MRD1 improved to 5 mm, and levator muscle function remained at 14 mm.

A summary of cases entering the study and treatment results are presented in Table 1.

The mean MRD1 among the studied patients improved from 1.58 mm before treatment to 3.66 mm 6 months after the treatment.

Subsequently, 6 months after the treatment, we encountered no complications in the studied patients, such as contour abnormalities, eyelid asymmetry and retraction, and dry eye syndrome.

## Discussion

In recent years, plasma application has been evaluated in treating dermatologic [9], gynecologic [10], and dental [11] disorders. Using plasma application has also been previously described in treating several ocular disorders such as conjunctival nevus, pinguecula, punctal occlusion, conjunctival cyst, and blepharoplasty with an acceptable safety profile [12–21].

A study by Rossi *et al*. [17] evaluated the clinical improvement and collagen remodeling of the upper eyelid dermatochalasis after plasma exeresis. Plasma exeresis in their pilot study showed promising remodeling effects on the collagen of the upper eyelid and clinically improved appearance for the patient cohort without any serious adverse events. Another study by Ferreira *et al*. [18] assessed patient satisfaction and symptoms after upper eyelid blepharoplasty with plasma technology.

Traditional surgical treatments for ptosis, such as levator muscle resection and advancement, are invasive techniques that might lead to eyelid contour abnormalities, asymmetry, and dry eye syndrome [22]. Ptosis surgery is recognized for its considerable complexity, and the outcomes can often be uncertain, resulting in either insufficient correction or excessive correction [23].

Recently, ptosis correction was performed using conjunctivo-mullerectomy, which consisted of theoretical excision of Muller’s muscle and the overlying conjunctive. Conjunctivo-mullerectomy is reserved for mild-to-moderate ptosis with good levator function, and some authors suggested this procedure in severe ptosis with positive phenylephrine test [18].

The basis of Muller’s muscle conjunctival resection (MMC) surgery is the removal of tissue, including the Muller muscle, conjunctiva, and part of the levator aponeurosis fibers. Plasma directly converts the targeted tissue from a solid to gaseous state and removes it from the surgical environment.

The mechanism of effect of this surgery has not been clearly determined since there is no direct relationship between the degree of eyelid elevation and the amount of muscle resection. It seems that in addition to the mechanical effect on muscle, other issues, such as adaptive neural processing mechanisms, are also involved [24].

This study examined a new approach to treating mild-to-moderate ptosis utilizing plasma-assisted Muller’s muscle sublimation. To the best of our knowledge, the present case series describes the first use of this method. The plasma-assisted method employed in this study offered the benefit of being a simple and noninvasive procedure, with satisfactory results achieved in a short follow-up period.

This study observed significant improvement in the MRD1 for all studied patients after applying plasma-assisted Muller’s muscle sublimation. These findings suggest that the direct application of plasma technology to Muller’s muscle as a minimally invasive approach might offer functional and aesthetic improvements for individuals affected by mild-to-moderate ptosis.

This study’s approach capitalized on the advantages of plasma energy, including precise tissue sublimation and shrinkage without the need for incisions or sutures, the short time needed to apply the treatment, and the advantage of giving the practitioner control over the treatment outcome.

Notably, the procedure took less than 5 minutes per eyelid in each treatment session for one lid, underscoring the time efficiency of this technique and its potential for widespread clinical application compared with surgical approaches. In addition, we could divide the treatment into multiple sessions to estimate each patient’s reaction to the first treatment session and continue further treatment on the basis of this reaction, thus lessening the chance of over- or undercorrection.

Using a low-temperature plasma device, such as a Plexr, can effectively support the health of conjunctival cells. This device aids in repairing conjunctival tissue around the sublimation site, promoting healing without causing scarring or leading to dry eye [10].

Nejat *et al*. reported that pinguecula treatment using atmospheric low-temperature plasma did not result in significant changes in dry eye tests [13].

However, this study has limitations. The small sample size and the absence of a control group undergoing traditional surgical treatments limit the ability to draw comprehensive conclusions from the data. Including control groups in future studies could provide a more apparent comparative analysis between plasma-assisted and traditional surgical approaches. Future research should aim to track outcomes over a more extended period to determine the durability of the treatment results.

Plasma technology applied to treating mild-to-moderate ptosis via Muller’s muscle sublimation seems to be a promising alternative to more invasive surgical methods, offering a rapid, simple, and effective solution for patients. Further studies with larger sample sizes, a control group, and longer follow-up periods are essential to validate these findings and fully establish this approach’s clinical efficacy and safety.

## Conclusion

By sublimating the conjunctivo-Muller (a novel technique) via plasma therapy, we were able to achieve significant results without any complications in a very short time and without the need for invasive mullerectomy surgery. This study can be the basis for future studies on this easy and noninvasive method.

## Data Availability

It will be available from the author upon request.
